# Reversible switching of magnetic states by electric fields in nitrogenized-divacancies graphene decorated by tungsten atoms

**DOI:** 10.1038/srep07575

**Published:** 2014-12-19

**Authors:** Gui-Xian Ge, Hai-Bing Sun, Yan Han, Feng-Qi Song, Ji-Jun Zhao, Guang-Hou Wang, Jian-Guo Wan

**Affiliations:** 1 National Laboratory of Solid State Microstructures and Department of Physics, Nanjing University, Nanjing 210093, China; 2 Key Laboratory of Ecophysics and Department of Physics, College of Science, Shihezi University, Xinjiang 832003, China; 3 Key Laboratory of Materials Modification by Laser, Ion and Electron Beams (Dalian University of Technology), Ministry of Education, Dalian 116024, China; 4 Collaborative Innovation Center of Advanced Microstructures, Nanjing University, Nanjing 210093, China

## Abstract

Magnetic graphene-based materials have shown great potential for developing high-performance electronic devices at sub-nanometer such as spintronic data storage units. However, a significant reduction of power consumption and great improvement of structural stability are needed before they can be used for actual applications. Based on the first-principles calculations, here we demonstrate that the interaction between tungsten atoms and nitrogenized-divacancies (NDVs) in the hybrid W@NDV-graphene can lead to high stability and large magnetic anisotropy energy (MAE). More importantly, reversible switching between different magnetic states can be implemented by tuning the MAE under different electric fields, and very low energy is consumed during the switching. Such controllable switching of magnetic states is ascribed to the competition between the tensile stain and orbital magnetic anisotropy, which originates from the change in the occupation number of W-5d orbitals under the electric fields. Our results provide a promising avenue for developing high-density magnetic storage units or multi-state logical switching devices with ultralow power at sub-nanometer.

Magnetic nanostructures with giant magnetic anisotropy energy (MAE) are of great interest for developing high-density spintronic data storage devices with the reduction of bit size^1^,^2^,^3^,^4^,^5^. Nevertheless, the great competition lies between the MAE and the energy consumption because a large value of MAE means that high magnetic fields are required to write bit information, which is quite energy-consuming^6^,^7^. Thus it is important to hunt for an available way to tune the MAE of magnetic units during writing or reading the information. In previous investigations, the electric field control has been proposed as a promising method to achieve the MAE switching^8^,^9^,^10^,^11^,^12^,^13^,^14^,^15^,^16^,^17^,^18^,^19^. It is then crucial to search for a stable material system with both large MAE and high sensitivity to electric fields. Recent studies suggest that graphene-based materials are good candidates due to their unique electronic structures and superior mechanical strength^20^,^21^,^22^,^23^. The hybridization between transition metal (TM) atoms (e.g. Au) and monolayer graphene can produce large MAE; meanwhile, the MAE value can be manipulated by external electric fields^24^,^25^. However, owing to very small energy barrier, the TM atoms prefer to glide randomly on the surface of graphene (and then aggregate) rather than stay on their original positions^26^,^27^,^28^. From the viewpoint of actual applications, such unstable hybrid system can hardly be used in magnetic storage units.

The stability of the TM atoms on graphene can be greatly enhanced if they are absorbed on the graphene with defects. Our recent work shows that defective graphene containing single vacancies (SVs) can anchor the TM atoms firmly at the centers of SVs while the MAE of the system remains large^23^. Another available option is to introduce divacancy defects such as nitrogenized divacancies (NDVs) into the graphene^29^. Since nitrogen atom has one more electron than carbon atom, both the surface activity and electronic structure of the graphene can be modified, thereby providing a possibility to improve the stability of the hybrid system. Moreover, the interaction between nitrogen atoms and TM atoms allows us to modulate the electronic structures of the whole system.

In this work, based on the first-principles calculations we demonstrate that the hybridization between the NDV-graphene and tungsten atoms can result in both high stability and large MAE for the W@NDV-graphene, meanwhile the whole hybrid system exhibits facile manipulation of MAE by external electric fields. We show that the conversion between different magnetic states in the system can be easily realized by controlling the switching of intermediate magnetic state upon the application of moderate electric fields. Such conversion of magnetic states hardly consumes energy, providing us a promising avenue to develop high-density magnetic storage units or multi-state logical switching devices with ultralow power at sub-nanometer. We also give an insight about the origin of such tunable MAE manipulated by external electric fields. We reveal that the change in the occupation number of W-5d orbitals under different electric fields plays an important role in determining the change in magnetic properties of the hybrid system, and the competition between the tensile stain and orbital magnetic anisotropy is responsible for the change in MAE under external electric fields.

## Results

### Structure and stability of W@NDV-graphene

**Figure 1:**
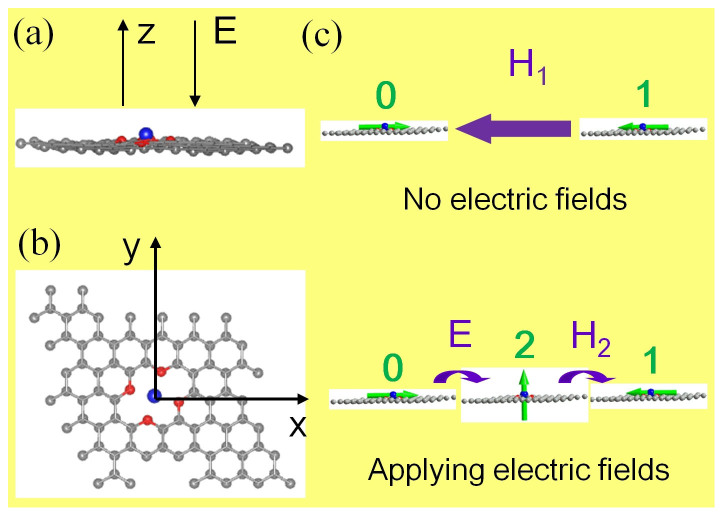
Structure of the W@NDV-graphene and schematic diagram of its application. (a,b) A side view and a top view, respectively. The gray, red and blue balls stand for C, N and W atoms, respectively. The electric field is oriented perpendicular to the graphene surface down-wards. The NDV-graphene was modeled by a 6 × 6 graphene supercell with a divacancy defect in which four carbon atoms around a divacancy was substituted by four nitrogen atoms. (c) Schematic illustration of the magnetic recording mode and multi-state logical switching using a W@NDV-graphene unit. The upper and lower illustrations show the magnetization reversals without and with external electric field (E), respectively. The green arrows represent magnetization direction, H_1_ and H_2_ represent the applied magnetic bias. “0”, “1”and “2” stand for three magnetic states.

Figures 1(a) and 1(b) present the stable structure of the W@NDV-graphene. In the configuration, the W atom locates exactly on the top of the NDVs. As displayed in Table 1, the height of the W atom to NDV-graphene plane is 0.507 Å, while the on-site charge of W atom is quite large, amounting to 1.519 e, as an indicative of charge transfer from W to C atoms. Such short distance and large charge transfer imply an enhanced interaction between the W atom and NDV-graphene, consequently stabilizing the adsorption of W atom on graphene. Our subsequent calculations of binding energies further confirmed that the W atom could stay on the top of the NDV-graphene very stably. Here we define the binding energy as 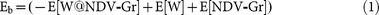
where E[W@NDV-Gr], E[W], and E[NDV-Gr] represent the total energies of W@NDV-graphene, W atom, and NDV-graphene, respectively. As listed in Table 1, the binding energy of the W@NDV-graphene reaches 7.83 eV, which is large enough to keep the W atom staying on the surface of NDV-graphene firmly. For comparison, if the W atom is absorbed on the perfect graphene, the whole system only has a much smaller binding energy (1.96 eV). The energy barrier for the migration of the W atom from one hollow site to another hollow site of the graphene is only 0.5 eV, implying that the W atom can easily slide on the perfect graphene even by a small temperature perturbation.

### Magnetic properties of W@NDV-graphene

**Figure 2:**
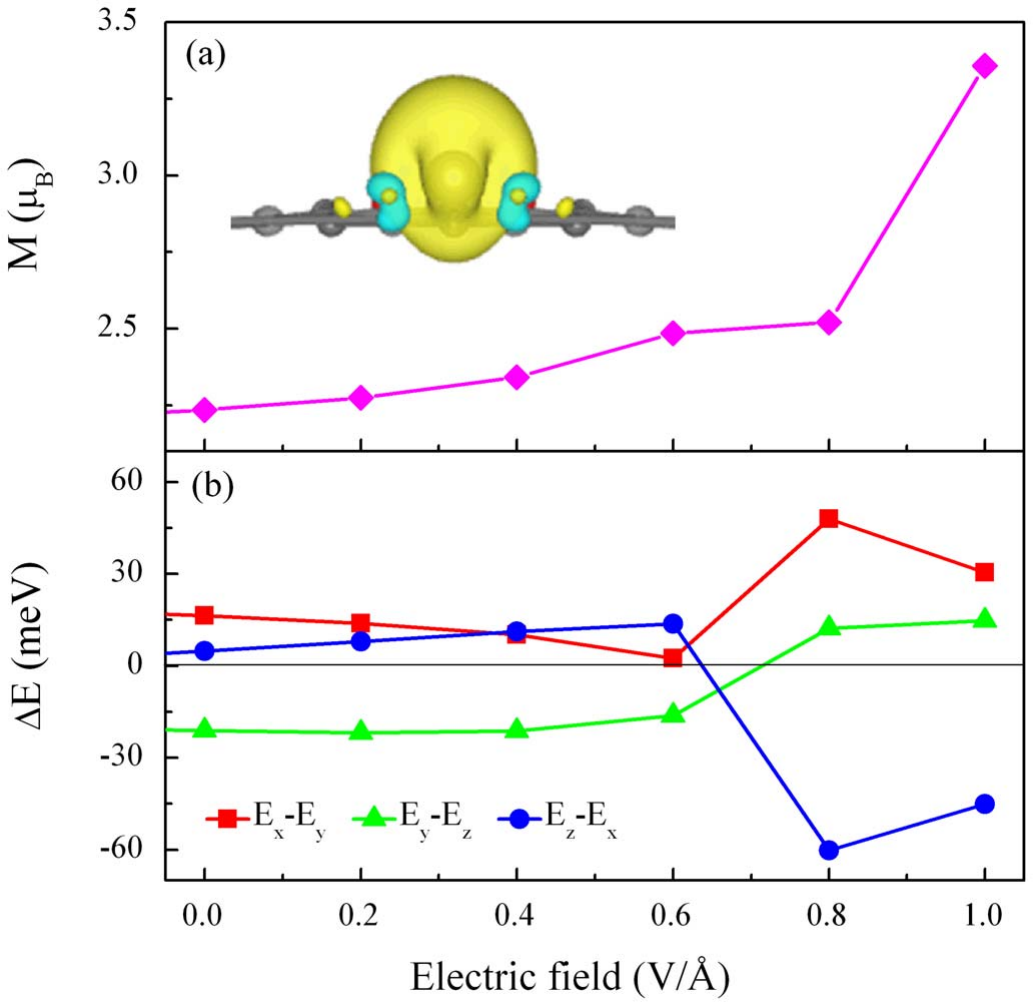
Variations of magnetic moment and MAE of the W@NDV-graphene with external electric fields. (a) Total spin magnetic moment as a function of electric field. The insert is the spin density of W@NDV-graphene. (b) Energy difference between each two magnetization directions of W@NDV-graphene under various electric fields.

Our calculations of magnetic moments show that the W@NDV-graphene possesses a total spin magnetic moment of 2.24 μ_B_. According to the spin density isosurface displayed in the inset of Figure 2(a), we find that the spin density of the W atom is much larger than those of N atoms. This indicates that the magnetic moment of the entire hybrid system mainly comes from the W atom. It is interesting that, doping N atoms into the graphene does not diminish the MAE of the whole system; on the contrary, the hybrid system of W@NDV-graphene exhibits a considerably large MAE of 21.17 meV. The value is much larger than that of W@DV-graphene (0.366 meV, here DV represents divacancy defect), also larger than that of W@graphene (about 16 meV)^20^. Based on the data listed in Table 1, we suggest that the enhanced MAE in the W@NDV-graphene originates from the increase in spin polarization of the W atom due to the incorporation of N atoms into graphene.

### Electric-field manipulation of MAE in W@NDV-graphene

To examine the feasibility of manipulating the MAE by external electric fields, we calculated the response of the magnetic properties of the W@NDV-graphene to the electric field. An electric field was applied to the W@NDV-graphene by the dipole layer method^30^. The electric field is oriented perpendicular to the graphene surface down-wards. Figure 2(a) displays the electric-field dependence of the total magnetic moment for the W@NDV-graphene. The total magnetic moment almost increases linearly with increasing the electric field up to 0.8 V/Å, then abruptly jumps to a high value of 3.35 μ_B_ at 1.0 V/Å. Figure 2(b) presents the variation of energy difference between each two magnetization directions with external electric fields. Before applying the electric field, the energy differences between each of the two magnetization directions are E_x_ − E_y_ = 16.36 meV, E_y_ − E_z_ = −21.17 meV, and E_z_ − E_x_ = −4.803 meV, respectively. Accordingly, the MAE of the hybrid system is determined to be 21.17 meV with an easy magnetization direction parallel to the graphene plane (along y axis) and a hard magnetization direction perpendicular to the graphene plane (along z axis). Applying external electric fields below 0.6 V/Å only gives rise to a perturbation of energy difference between each of the two magnetization directions, and the easy magnetization axis also does no change. Surprisingly, when the applied electric field is close to 0.8 V/Å, not only the energy difference between each of the two magnetization directions changes remarkably, but also the easy and hard magnetization axes are switched completely, i.e. the easy magnetization axis changes to z axis whilst the x axis becomes the hard magnetization axis. At 0.8 V/Å, the energy differences between each of the two magnetization directions become E_x_ − E_y_ = 47.979 meV, E_y_ − E_z_ = 12.284 meV, and E_z_ − E_x_ = −60.263 meV, respectively. The MAE of the whole system thus reaches a very high value of 60.26 meV, about three times of the original MAE value at zero electric field. When further increasing the electric field, the MAE only slightly decreases and both the easy and hard magnetization axes remain unchanged.

Both high stability and large MAE indicate that the W@NDV-graphene hybrid system can be used as a single-molecule-magnet-like unit for magnetic storage. Considering that the size of the W@NDV-graphene supercell used in this work is about 1.5 nm, if the W@NDV-graphene units are assembled on this scale, the spacing between each unit is large enough to avoid the coupling interaction with each other (see Supplementary Information for more details on the calculations). Based on the rule that the MAE value of the recording media should exceed 40k_B_T for 1-bit storage^31^, we estimate that the magnetic storage temperature for a single W@NDV-graphene unit with the MAE value of 21.17 meV can reach T_ms_ = 6.1 K, larger than those of most single-molecule magnets in the current stage such as Fe_4_-organometallic complexes (T_ms_ = 0.5 K)^32^, dysprosium-based endohedral single-molecule magnets (T_ms_ = 5.5 K)^33^. More importantly, its tunable MAE manipulated by external electric fields offers great opportunities to develop high-performance magnetic storage media with ultralow energy consumption, which is superior to most single-molecule-magnets. Figure 1(c) gives the schematic illustration of the recording mode using W@NDV-graphene as a magnetic storage unit. If no electric field is applied to the unit, in order to realize the magnetization conversion from initial “0” state to final “1” state, a high magnetic field has to be applied, which is energy-consuming. Differently, upon the application of moderate electric fields, the magnetic state can be easily converted from initial “0” state to intermediate “2” state by 90-degree switching of magnetization direction, and the subsequent magnetization reversal from intermediate “2” state to final “1” state will be easily realized if a very small magnetic field is exerted^34^,^35^. This process only consumes little power^36^. Such kind of hybrid system is also a good candidate for developing multi-state logical switching devices with low-power at sub-nanometer, e.g. the interconversion among “0”, “2” and “1” states with or without electric fields. In addition, the tunable MAE value under electric fields also allows us to further improve the stability of the magnetic states.

## Discussion

### Origin of tunable MAE by electric fields

**Figure 3:**
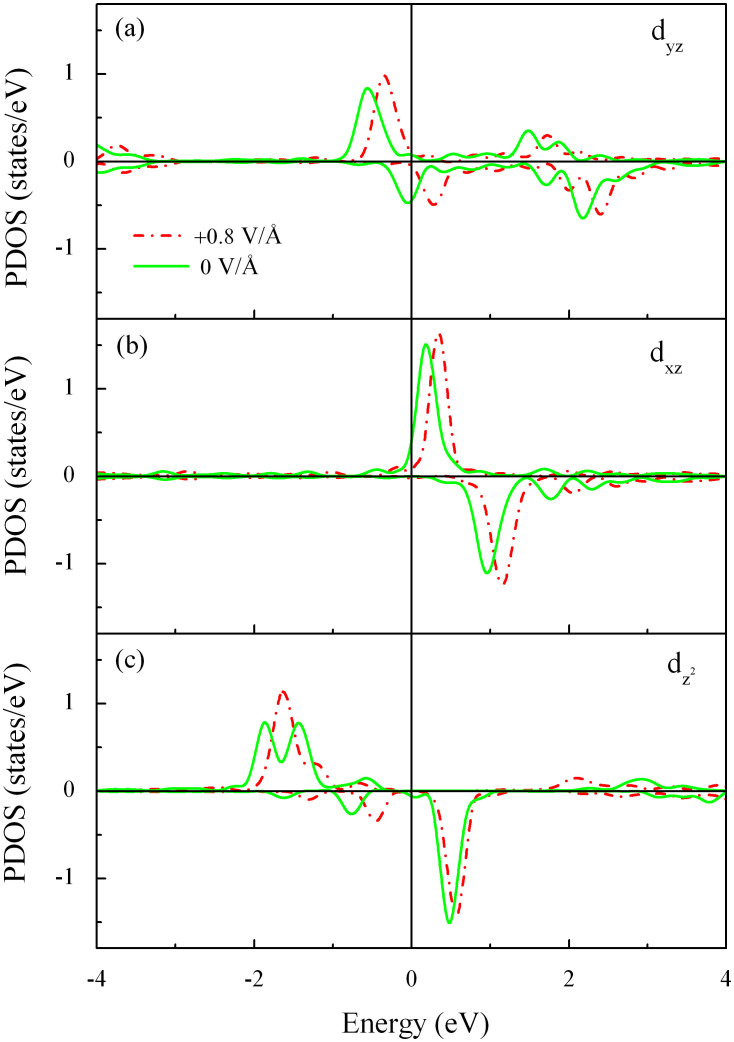
Change of projected density of states (PDOS) of typical W-5d orbitals in the W@NDV-graphene upon the application of +0.8 V/Å electric fields. (a) d_yz_ orbital, (b) d_xz_ orbital and (c) d_z_^2^ orbital.

We now turn to explore the mechanism of manipulating MAE by electric fields for the present hybrid system. The influence of the electric field on the occupation number of W-5d orbitals in the W@NDV-graphene was first considered. Figure 3 plots the projected density of states (PDOS) of W-5d orbitals under the electric fields of 0 and 0.8 V/Å. It is seen that most dominant states locate near the Fermi level (E_F_). When the electric field is increased from 0 to 0.8 V/Å, the PDOS of W-5d orbitals has a drastic change, indicating that the electric field induces variation of occupation number of W-5d orbitals. Based on the second perturbation theory^37^, the electric field modulation on the electronic structure around the E_F_ is related to the change of MAE as follows: 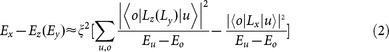
where o and u specify the occupied and unoccupied valance states, respectively, and L_x_, L_y_ and L_z_ are the angular momentum operators, respectively. According to Eq. (2), the most dominant contributions to the MAE come from the states near the E_F_, and its behavior is substantially determined by the denominator. We have calculated the spin orbital coupling (SOC) matrix elements between different d orbitals^38^, and found that only the d_yz_, d_xz_ and d_z_^2^ orbitals have large contributions to the MAE. Moreover, from the second term in Eq. (2). It is clear that the electric field affects the coupling between d_yz_ and d_z_^2^ orbitals dramatically through L_x_ operator, leading to the change in MAE.

**Figure 4:**
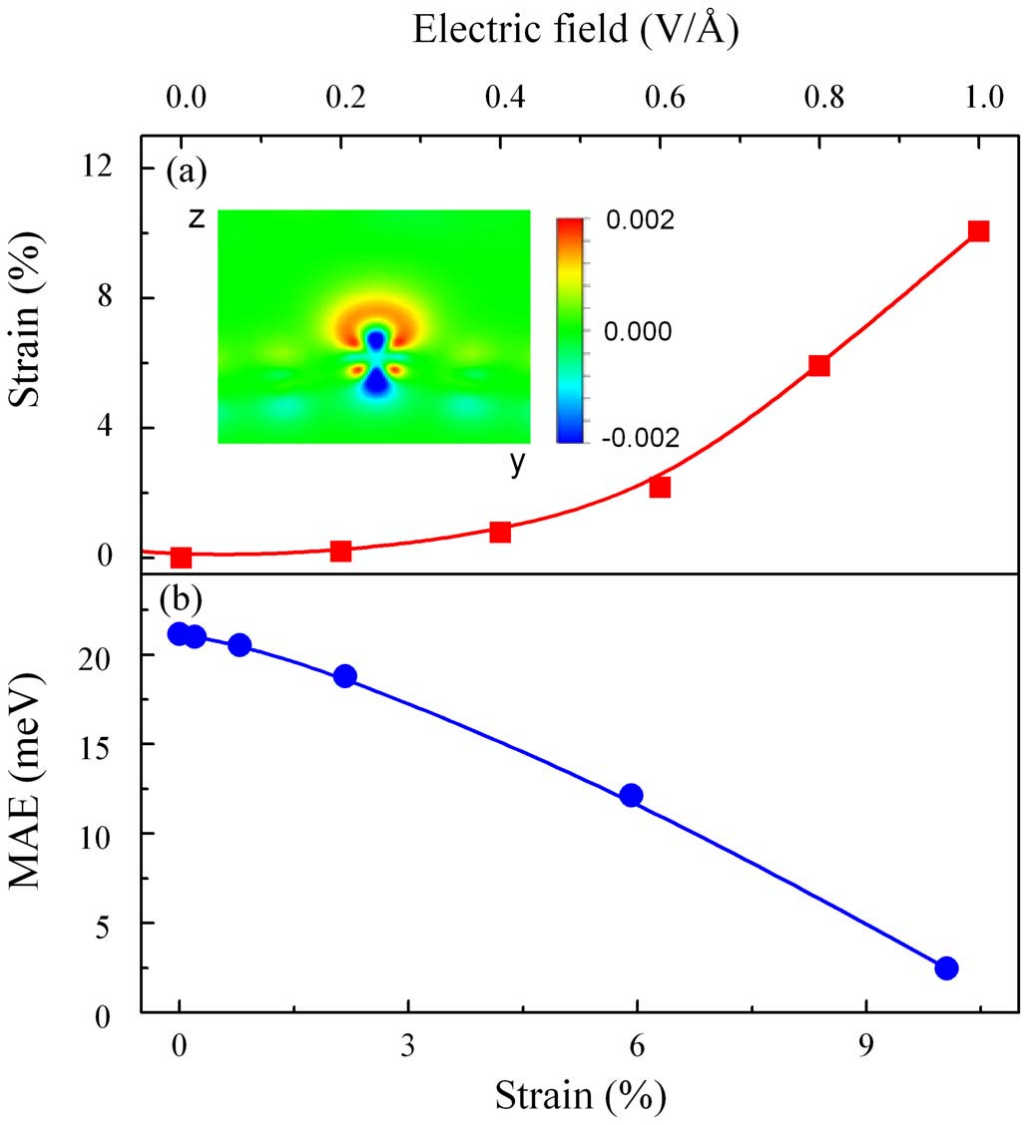
Influence of strain on the MAE of the W@NDV-graphene. (a) Strain as a function of electric field, (b) MAE value as a function of strain. The insert of (a) displays electric-field-induced charge rearrangement in yz plane after the hybrid system is under an electric field of 0.8 V/Å.

We can classify the contributions of PDOS to MAE into two groups, one involving the coupling between the same spin states and the other including the coupling between different spin states (i.e. the spin-flip terms). Previous investigations of FeCo/MgO(011) thin films have revealed that the spin-flip terms are much smaller when the exchange splitting between majority and minority states is large^39^. Since the W@NDV-graphene has large spin magnetic moment, we only consider the coupling between the same spin states. As displayed in Figure 3, without the external electric fields the most states near the E_F_ contribute to the MAE are d_z_^2^, d_yz_ and d_xz_. The magnetization direction is mostly determined by the SOC of up-spin d_z_^2^ with d_xz_ state through L_y_ operator, and then the easy axis is parallel to the graphene plane. When an external electric field is applied, the occupation number of the down-spin d_yz_ orbital of W atom is reduced, consequently shifting the d_yz_ orbital to the higher energy side. The reduced electron occupation number of d_yz_ orbital is also visualized by the charge-density difference between 0 and 0.8 V/Å, as plotted in the inset of Figure 4(a). Evidently, the electric field also causes the reduction of occupied up-spin and unoccupied down-spin d_z_^2^ orbital. As a result, the coupling interaction between down-spin d_yz_ and d_z_^2^ through L_x_ operator is reduced. In addition, both the occupied up-spin d_yz_ and unoccupied up-spin d_xz_ increase, indicating that the coupling interplay between d_yz_ and d_xz_ through L_z_ operator is enhanced while the coupling interplay between d_z_^2^ and d_xz_ is reduced due to the decrease of up-spin d_z_^2^ and the shift to higher energy side of up-spin d_xz_. Therefore, the MAE of the hybrid system increases and the magnetization direction of the easy axis changes.

### Competition between tensile strain and orbital magnetic anisotropy

Our further calculations show that applying electric fields to the W@NDV-graphene can cause an increase in the distance between the W atom and graphene plane (e.g. the increment reaches 0.537Å at 0.8 V/Å). This indicates that a tensile stain perpendicular to the graphene plane is induced. Figure 4(a) plots the strain perpendicular to the graphene plane as a function of electric field. The induced tensile strain increases with increasing the applied electric field, subsequently giving rise to a change in MAE, as shown in Figure 4(b). For instance, when the electric field increases from 0 to 1.0 V/Å, the tensile strain increases from 0 to 10%, accompanying with an evident decrease of MAE from 21.17 to 2.36 meV. This means that the tensile strain induced by the electric field actually has the negative influence on the overall increased MAE of the hybrid system.

**Figure 5:**
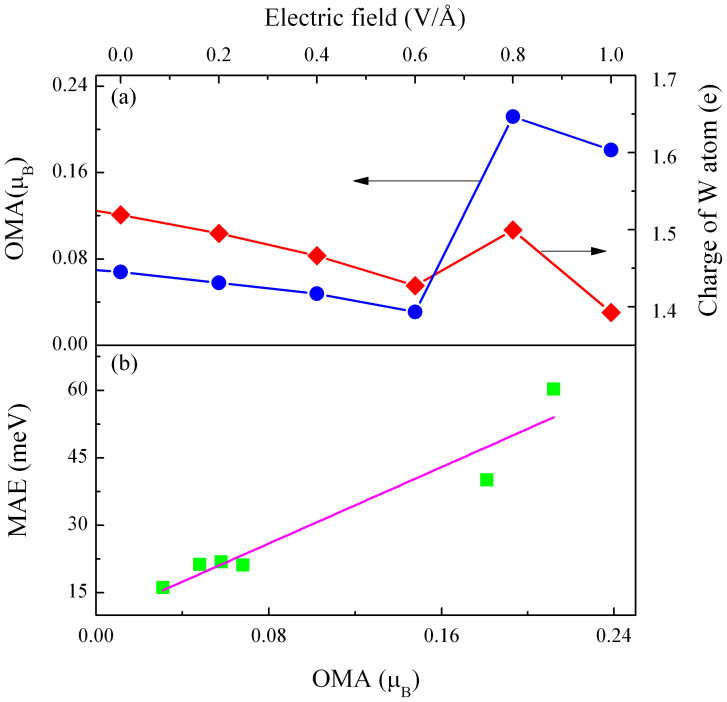
Influence of orbital magnetic anisotropy (OMA) on the MAE of the W@NDV-graphene. (a) Variations of OMA and charge of W atom with electric fields, (b) MAE as a function of OMA.

Meanwhile, we find that external electric fields can also cause the charge rearrangement of W atom on the surface of NDV-graphene. Previous investigations of Fe bilayer in Pt/Fe_2_/Pt(100) have revealed that the charge transfer has great influence on the orbital magnetic anisotropy (OMA, i.e. the difference in orbital magnetic moment between easy and hard magnetization directions)^40^. Since the MAE of the magnetic system is sensitive to the OMA which is susceptible to the charge transfer, we calculated the change in both the charge of W atom and the OMA under various electric fields, as shown in Figure 5(a). The charge of W atom varies in the range from 1.52 to 1.39 e, depending on the applied electric field, meanwhile, the OMA value fluctuates between 0.03 and 0.21 μ_B_. According to Bruno's relationship^41^, the MAE is generally proportional to the OMA in a magnetic system as follows: 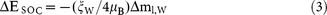
where ξ_W_ is an average value of the SOC coefficients of W atom, and Δm_l,W_ is the difference of orbital magnetic moment between the easy and hard magnetization directions. Our results shown in Figure 5(b) demonstrate that the MAE almost exhibits a linear dependence on the OMA for the W@NDV-graphene when it is under external electric fields, complying with the Bruno's relationship. The Bruno's relationship is valid only when the exchange splitting is sufficiently larger than the bandwidth, i.e. when the hybridization is weak^6^. For the present hybrid system, before applying the electric field, the spin magnetic moment of whole system reaches a larger value (2.24 μ_B_), indicative of a sufficient exchange splitting of the W atom. After applying external electric fields, the reduction in charge of W atom leads to an increase in full spin magnetic moments (seen in Figure 2(a)), further resulting in an enhancement of exchange splitting of the W atom.

Accordingly, we propose that the change in MAE of the W@NDV-graphene under external electric fields is actually associated with the competition between the tensile stain and OMA in the system. When the applied electric field is lower, the increase of MAE caused by OMA overtakes the decrease of MAE caused by tensile stain. As a result, the total MAE of the system increases with increasing the electric field. The situation becomes different when the W@NDV-graphene is under higher electric fields (beyond 0.8 V/Å). At high electric fields, the suppression of MAE by tensile strain becomes stronger than the increase of MAE caused by OMA, causing the drop of total MAE. Evidently, from the view of applications, the MAE manipulation by electric fields is more efficient if the hybrid system is under stress-clamping state.

Overall, we have demonstrated that the amplitude of MAE and magnetization direction of the W@NDV-graphene can be facilely manipulated by external electric fields. The hybrid system has both high stability and large MAE value. The change in the occupation number of W-5d orbitals under electric fields plays an important role in determining the change in magnetic properties of the hybrid system. Applying electric fields can cause the change in both tensile stain and orbital magnetic anisotropy. The competition between these two parameters determines the change in MAE under electric fields. Considering the tunable MAE manipulated by external electric fields allows us to largely reduce the energy consumption during operating the magnetic states, we expect such kind of hybrid system is promising for developing high-performance magnetic storage and multi-state logical switching devices with ultralow energy consumption at sub-nanometer scale.

## Methods

The first-principles calculations were performed in the framework of spin-polarized density functional theory (DFT) using the projector augmented wave (PAW)^42^,^43^ as implemented in the Vienna ab initio simulation package (VASP) code^38^. The exchange-correlation interactions were described with a generalized gradient approximation (GGA) in the form of the Perdew, Burke, and Ernzerh of (PBE) functional^44^. A 3 × 3 × 1 Γ-centered k-point mesh was used to sample the Brillouin Zone of the supercell. All geometric structures were fully relaxed using the conjugate gradient algorithm until the force on each atom was smaller than 0.01 eV/Å. The MAE value was evaluated by implementing spin-orbit coupling (SOC) in VASP in a noncollinear mode^45^. During the calculation of MAE, the geometric, electronic, and magnetic degrees of freedom were relaxed simultaneously until the change in total energy between successive iteration steps was smaller than 10^−7^ eV. The NDV-graphene was modeled by a 6 × 6 graphene supercell with a divacancy defect, in which four carbon atoms around a divacancy was substituted by four nitrogen atoms. To avoid interactions between the layers, vacuum region of 15 Å in the direction normal to graphene was employed.

## Author Contributions

G.X.G. performed the theoretical calculation. J.G.W. conceived and provided advice on the analysis. J.G.W., G.X.G. and Y.H. wrote the paper. H.B.S., F.Q.S., J.J.Z. and G.H.W. participated in analysis and discussion. All authors participated in the data discussion.

## Supplementary Material

Supplementary Information Supplementary Information

## Figures and Tables

**Table 1:** The distance (d) between the W atom and graphene, binding energy (E_b_), magnetic moment (M)^a^, the charge (Q)^b^ of atoms, and magnetic anisotropy energy (MAE) for W@NDV-graphene and W@DV-graphene

			Magnetic moment (μ_B_)			Charge (e)			
Hybrid System	d (Å)	E_b_ (eV)	M_total_	M_C_	M_N_	Q_W_	Q_C_	Q_N_	MAE (meV)
W@NDV-Graphene	0.508	7.83	2.24	0.015	−0.031	1.519	0.380	−1.195	21.17
W@DV-Graphene	0.819	8.557	0.47	−0.017	-	1.340	−0.246	-	0.366

^a^The magnetic moment (M) refers to the total system (M_total_), average magnetic moment of C near N atoms (M_C_), and N atoms (M_N_).

^b^The charge (Q) of atoms represents the charge of W atom (Q_W_), the average charge of C near N atoms (Q_C_), and N atoms (Q_N_).
