# Supplementary material for: Reversible switching of magnetic states by electric fields in nitrogenized-divacancies graphene decorated by tungsten atoms
Source: Sci Rep. 2014 Dec 19;4:7575. doi: 10.1038/srep07575 (PMC4271256; doi:10.1038/srep07575)
Supplement: Supplementary Information [file srep07575-s1.doc]

Supplementary Information

Reversible switching of magnetic states by electric fields in nitrogenized-divacancies graphene decorated by tungsten atoms

Gui-Xian Ge^1, 2^, Hai-Bing Sun^1^, Yan Han^1^, Feng-Qi Song^1,4^, Ji-Jun Zhao^3^, Guang-Hou Wang^1,4^, & Jian-Guo Wan^1,4^

^1^ National Laboratory of Solid State Microstructures and Department of Physics, Nanjing University, Nanjing 210093, China

^2^ Key Laboratory of Ecophysics and Department of Physics, College of Science, Shihezi University, Xinjiang 832003, China

^3^ Key Laboratory of Materials Modification by Laser, Ion and Electron Beams (Dalian University of Technology), Ministry of Education, Dalian 116024, China

^4^ Collaborative Innovation Center of Advanced Microstructures, Nanjing University, Nanjing 210093, China

Correspondence and requests for materials should be addressed to J.G.W. (email: [wanjg@nju.edu.cn](mailto:wanjg@nju.edu.cn))

**Calculations of indirect exchange coupling between W@NDV-graphene units:**

To study the indirect exchange coupling between the W@NDV-graphene units, we constructed a 6×9 supercell with two W atoms on NDV-graphene. In this configuration, the distance between the W atoms is 9.8 Å, as shown in Fig. S1. To avoid the interaction between the graphene layers, a vacuum layer of 15 Å was added along the graphene plane normal. The calculations were performed in the framework of spin-polarized density functional theory (DFT) using the projector augmented wave (PAW) as implemented in the Vienna ab initio simulation package (VASP) code. The exchange-correlation interactions were employed with a generalized gradient approximation (GGA) in the form of the Perdew, Burke, and Ernzerh of (PBE) functional. A 3 × 3 × 1 Γ-centered k-point mesh was used to sample the Brillouin Zone of the supercell.


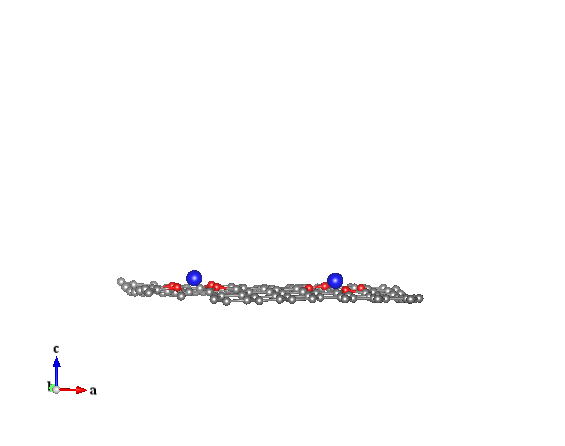


(a)


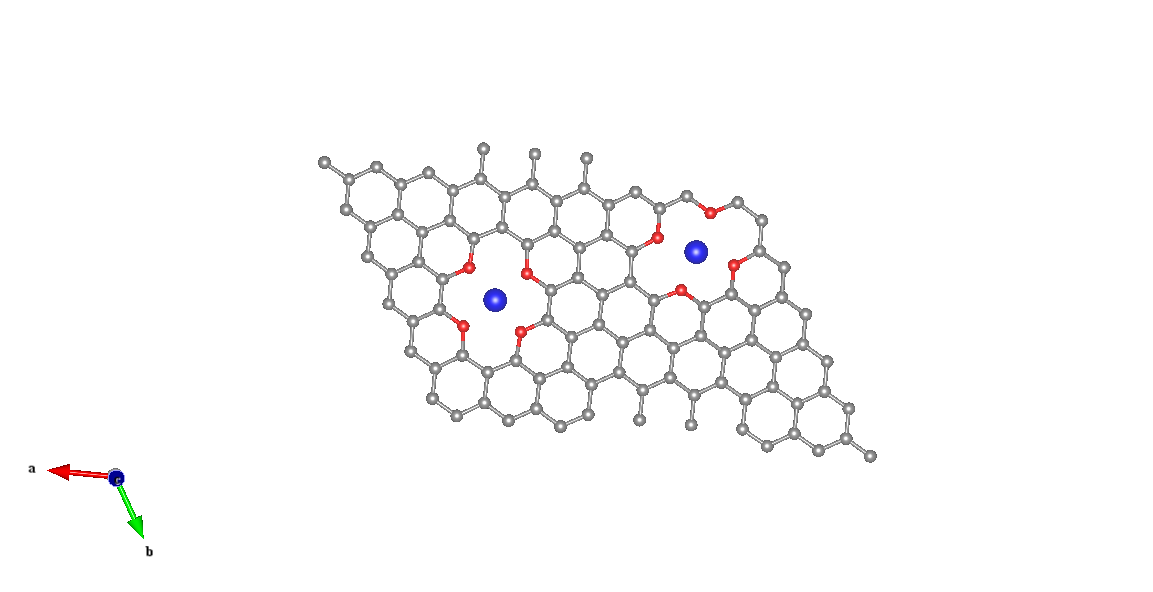


(b)

Fig. S1 A 6×9 supercell with two W@NDV-graphene units. (a) side view, (b) top view. The gray, red and blue balls stand for C, N and W atoms, respectively. The distance between the W atoms is 9.8 Å.

We estimated the strength of RKKY-type exchange coupling between the W@NDV-graphene units by calculating the total-energy difference between spin-parallel (ferromagnetic coupling) and spin-antiparallel (antiferromagnetic coupling ) configurations: [1]

ΔE = E_FM_ − E_AFM_ (S1)

The calculation results show that the ΔE value is only 5.6×10^-4^ eV when the distance between the W atoms is 9.8 Å, indicating that the RKKY-type interaction between the two units is rather weak.

We also plotted the distribution of spin charge density in the whole system, as shown in Fig. S2. The spin charge densities are highly localized around each W atom, further implying that the exchange coupling between the W@NDV-graphene units hardly happens.

**
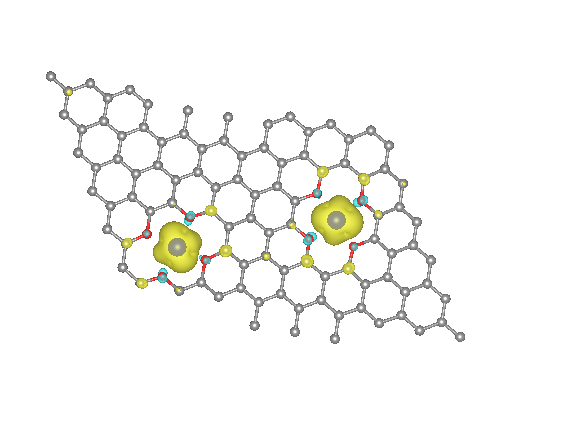

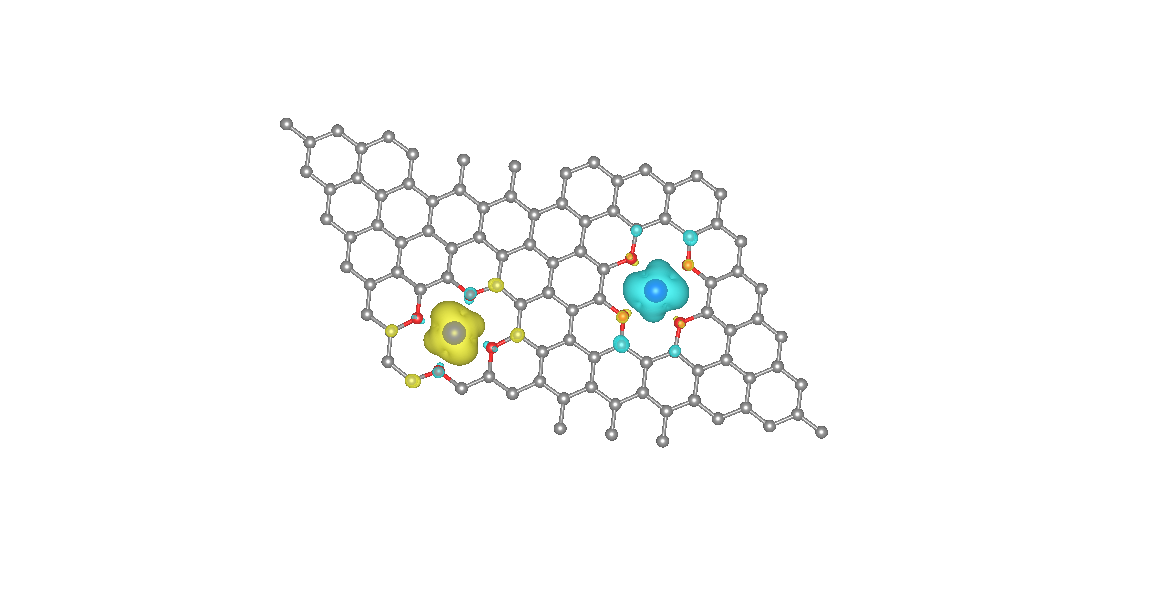
**

（a） (b)

Fig. S2 Distribution of spin-polarized charge density in the whole system. (a) Ferromagnetic coupling, (b) Antiferromagnetic coupling. The isosurface correspond to a value of 0.006 eÅ^-3^.

**References:**

[1] Chen, H., Niu, Q., Zhang, Z. Y. & MacDonald, A. H. Gate-tunable exchange coupling between cobalt clusters on graphene. *Phys. Rev. B* **87**, 144410 (2013).
